# Autophagy Proteins in Phagocyte Endocytosis and Exocytosis

**DOI:** 10.3389/fimmu.2017.01183

**Published:** 2017-09-22

**Authors:** Christian Münz

**Affiliations:** ^1^Viral Immunobiology, Institute of Experimental Immunology, University of Zürich, Zürich, Switzerland

**Keywords:** major histocompatibility complex, LC3-associated phagocytosis, IL-1, Epstein–Barr virus, varicella zoster virus, poliovirus, coxsackievirus

## Abstract

Autophagy was initially described as a catabolic pathway that recycles nutrients of cytoplasmic constituents after lysosomal degradation during starvation. Since the immune system monitors products of lysosomal degradation *via* major histocompatibility complex (MHC) class II restricted antigen presentation, autophagy was found to process intracellular antigens for display on MHC class II molecules. In recent years, however, it has become apparent that the molecular machinery of autophagy serves phagocytes in many more membrane trafficking pathways, thereby regulating immunity to infectious disease agents. In this minireview, we will summarize the recent evidence that autophagy proteins regulate phagocyte endocytosis and exocytosis for myeloid cell activation, pathogen replication, and MHC class I and II restricted antigen presentation. Selective stimulation and inhibition of the respective functional modules of the autophagy machinery might constitute valid therapeutic options in the discussed disease settings.

## Introduction

Autophagy is a group of at least three pathways that deliver cytoplasmic constituents for lysosomal degradation (1). While microautophagy and chaperone-mediated autophagy directly operate at the late endosomal or lysosomal membrane for cytosolic substrate engulfment or translocation, respectively, macroautophagy assembles double-membrane surrounded vesicles *de novo*, which are then transported to lysosomes. For this autophagosome generation and delivery to lysosomes, autophagy-related gene (*atg*) products are essential, the first 15 of these were identified by Yoshinori Ohsumi in 1993 (2) and formed the basis of the molecular machinery of macroautophagy that led to his Nobel Prize in 2016. These Atgs are organized in complexes that integrate metabolic cues to regulate macroautophagy and modify membranes by lipid phosphorylation and ubiquitin-like protein conjugation to lipids, which result in autophagosome formation and substrate recruitment. The Atg1/ULK1 complex is regulated through phosphorylation by mammalian target of rapamycin (mTOR) inhibition and AMP-activated protein kinase (AMPK) activation. These two pathways sense nutrient or growth factor depletion *via* decreased mTOR activity and low-energy levels, resulting in elevated AMP concentration, and *via* increased AMPK activity. Atg1/ULK1 in turn phosphorylates Atg6/Beclin-1, a regulatory subunit of the VPS34 type III phosphatidylinositol 3-kinase (PI3K) complex. The resulting phosphoinositide mark on membranes serves as the landing platform for WIPI proteins that recruit *via* Atg16L1 binding the machinery to conjugate Atg8/LC3 to phosphatidylethanolamine, which might mediate both the fusion of additional membranes to this site for double-membrane elongation to a cup-shaped isolation membrane, resulting in fusion of these double membranes to autophagosomes, and substrate recruitment into the autophagosome (3–6).For this purpose, yeast Atg8 and its six mammalian orthologs LC3A, B, C, GABARAP, GABARAP-L1, and GABARAP-L2 are first processed by Atg4 to expose a *C*-terminal glycine for the ubiquitin-like conjugation reaction, which is then executed by the E1-like enzyme Atg7, the E2-like enzyme Atg3 and the E3-like enzyme Atg12-Atg5/Atg16L1. This enzymatic cascade leads to Atg8/LC3 coupling to the outer and inner autophagosome membrane. While some of these Atg8 orthologs have membrane fusion activity on their own, they recruit substrates often through intermediaries that contain LC3-interacting regions (LIRs) (6). These include proteins that get exposed on damaged organelles, such as mitochondria (7), and others that bridge ubiquitinated substrates, such as protein aggregates and cytosolic bacteria with LC3 (8, 9). The latter include sequestosome/p62, NBR1, NDP52, and optineurin and are often investigated as prototypic macroautophagy substrates. The completed autophagosome loses much of its LC3 from the outer membrane by deconjugation by Atg4, but retains some to facilitate transport along microtubules *via* FYCO1 and NDP52 recruitment (10, 11) and lysosome fusion *via* binding to PLEKHM1 (12). The much higher affinity of the PLEKHM1 LIR for GABARAPs might, however, indicate that these cytosolic functions are executed by Atg8 orthologs that do not belong to the LC3 subfamily (13). HOPS complex and Rab7 recruitment then prepare for lysosome fusion, which is executed by the SNAREs syntaxin17, SNAP29, and VAMP8 (14). This leads to lysosomal degradation of not only the autophagosome cargo but also the inner autophagosomal membrane including the Atg8/LC3 molecules that are still coupled to it. Therefore, Atg8/LC3 turnover, especially of its lipidated form LC3-II, serves also as a measure of macroautophagy. This modular format of the macroautophagy machinery lends itself to membrane modifications during cell biological processes that are distinct from macroautophagy. For example, the cascade of ULK1 and VPS34 complexes can put phosphoinositide marks on non-isolation membranes and the cascade of VPS34 and Atg8 lipidation complexes can label non-autophagosomal membranes with Atg8/LC3 (15, 16). While these modules are successively used by macroautophagy to restrict intracellular pathogens, like bacteria and viruses (17–19), and to degrade intracellular proteins for major histocompatibility complex (MHC) class II restricted antigen presentation, during anti-viral immune responses and CD4^+^ T cell education (20, 21), individual modules are used in alternative pathways, including proviral roles in infectious viral particle release, restriction of phagocytosed bacteria, secretion of inflammatory mediators, and presentation of phagocytosed antigens on MHC molecules (22–29). The characteristics and functional roles of the respective pathways will be discussed in this minireview.

## Atg Proteins in LC3-Associated Phagocytosis (LAP)

**Figure 1:**
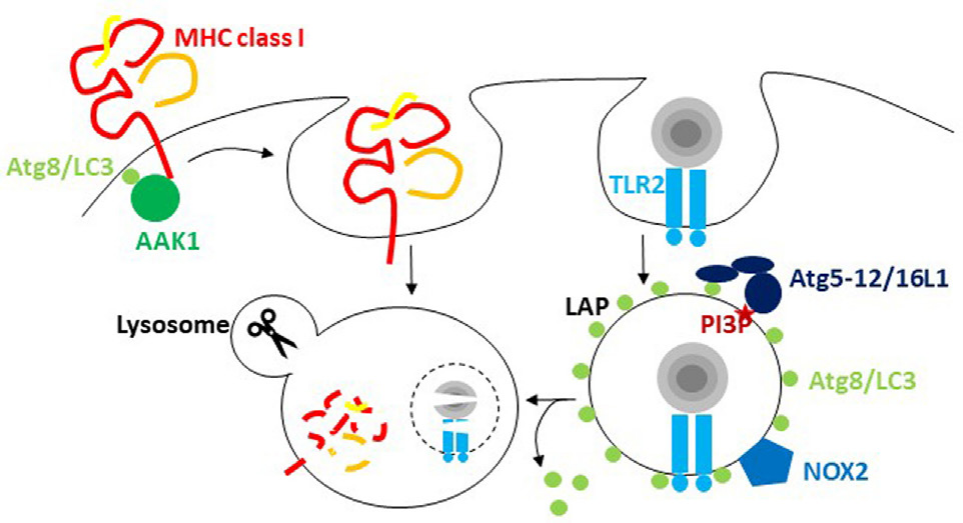
The macroautophagy machinery regulates endocytosis. Atg8/LC3 lipidation facilitates the internalization of receptors from the cell surface *via* recruitment of components of clathrin-mediated endocytosis. For major histocompatibility complex (MHC) class I internalization, recruitment of adaptor-associated kinase 1 (AAK1) facilitates MHC class I internalization and degradation in lysosomes. During LC3-associated phagocytosis (LAP), endocytosed cargo that engages receptors like TLR2 stimulates the conjugation and/or maintenance of Atg8/LC3 lipidation on the cytosolic side of the phagosome. PI3 phosphorylation recruits the Atg8/LC3 conjugation machinery, including Atg5, 12, and 16L1, to these phagosomes, and NADPH oxidase 2 (NOX2)-dependent reactive oxygen species (ROS) production is required for LAP. Atg8/LC3 conjugation to phagosomes regulates their fusion with lysosomes.

The most prominent of these alternative pathways is probably LAP. It was originally reported in 2007 that Atg8/LC3 can also be conjugated to phagosomal membranes, especially after the uptake of particulate toll-like receptor (TLR) ligands (Figure 1) (25). For example, the yeast cell wall component zymosan is often used for these assays (25, 29, 30). Apart from TLRs, a handful of other receptors seem to trigger LAP. These include the C-type lectin Dectin-1, Fc receptors during the uptake of antibody opsonized targets and receptors for apoptotic whole cells or cell fragments (30–33). During LAP, Atg8/LC3 gets conjugated to the cytosolic side of the phagosomal membrane and dissociates before phagosome fusion with lysosomes (25, 29). The VPS34 complex including Beclin-1 and the Atg lipidation machinery but not the ULK1 complex is required for this Atg8/LC3 lipidation (29, 34). Instead reactive oxygen species (ROS) production by the NADPH oxidase 2 (NOX2) is either required for Atg8/LC3 lipidation or maintenance of Atg8/LC3 on the phagosomal membrane (29, 34). This also probably explains earlier findings that suggested ROS production by NOX2 being required for the recruitment of autophagosomes to endocytosed *Salmonella* bacteria (24). Furthermore, Rubicon, a negative regulator of autophagosome fusion with lysosomes, seems to be required for LAP (34, 35). In contrast to the role of Rubicon during autophagosome maturation, LAP vesicles have been reported to fuse with lysosomes more rapidly than LC3-negative phagosomes in mouse macrophages (25, 34, 36). This enhanced maturation might result from accelerated transport along microtubules *via* FYCO1 recruitment by Atg8/LC3 binding (36). However, in other cell types, namely human macrophages as well as conventional and plasmacytoid dendritic cells (DCs), LAP phagosomes might not rapidly fuse with lysosomes, but rather retain phagocytosed cargo for delayed delivery to lysosomes and to endogenous TLR, like TLR9, containing vesicles (29, 31). However, why and how phagosomes use the Atg8/LC3 membrane tag to regulate their phagosome trafficking needs further investigations. This regulation seems to increase MHC class II restricted antigen presentation and to decrease inflammation. LAP deficiency compromised extracellular antigen presentation on MHC class II molecules to CD4^+^ T cells (29, 30). Also *in vivo*, CD4^+^ T cell responses to herpes simplex virus (HSV) infection and ovalbumin containing apoptotic splenocyte injection were compromised in the absence of Atg5 in DCs (28). In addition, cross-presentation of antigens of *Aspergillus, Chlamydia*, and human cytomegalovirus on MHC class I molecules was found to be inhibited by Atg deficiency (37–39). In macrophages, the dominant phenotype of Atg deficiency is a hyperinflammatory phenotype (40), probably originating to a large extent from mitochondrial ROS-mediated inflammasome activation in the absence of macroautophagy of damaged mitochondria (41). Interestingly, aging of mice with macrophage deficiencies of Atg7, Atg5, Beclin-1, NOX2, or Rubicon developed signs of hyperinflammatory disease, while this phenotype was far less pronounced in mice with ULK1 and FIP200 deficiencies in macrophages (35). These findings suggested that LAP, but not classical macroautophagy, protects wild-type mice from this aging-related hyperinflammation. In addition, the development of lupus-like anti-DNA immune complex deposition in kidneys and elevated pro-inflammatory cytokine titers were detected. Surprisingly, the increase in inflammasome-dependent IL-1β production was quite pronounced, but the mechanism of LAP-mediated inflammasome regulation remains unclear. Instead, deficient LAP-mediated apoptotic cell clearance might be mainly responsible for the observed hyperinflammatory phenotypes, and indeed, the investigated parameters were similar to mice lacking apoptotic cell clearance due to deficiency of the TIM4 receptor in their macrophages. Thus, the VPS34 and LC3 lipidation complexes of the macroautophagy machinery seem to modify phagosomes for improved antigen presentation and inhibition of hyperinflammation.

## Atg Proteins in Receptor Internalization and MHC Class I Antigen Presentation

In addition to this role of Atg8/LC3 lipidation in influencing phagosome fate, recent studies have suggested that recruitment of the receptor internalization machinery can also benefit from Atg8/LC3 binding (Figure 1). In pioneering studies, Alzheimer precursor protein (APP) was shown to be degraded by an ULK1, Atg6/Beclin-1, and Atg5-dependent mechanism (42). The internalization of APP that is required for this degradation is mediated by clathrin-dependent phagocytosis, which requires the adaptor protein 2 (AP2) complex. AP2α1 was identified as a Atg8/LC3 interactor by the same group (43). It contains a LIR motif, and mutating it abolished efficient APP internalization and degradation. Furthermore, phosphorylation or the APP-degrading enzyme presenelin 1 facilitated APP degradation, possibly by syntaxin 17-mediated fusion with lysosomes (44, 45). Thus, Atg8/LC3-mediated AP2 recruitment and syntaxin 17-mediated fusion with lysosomes seem to cause efficient degradation of APP and its *C*-terminal fragment from the cell membrane. However, AP2 recruitment to Atg8/LC3 does not seem to be the only connection of the autophagic machinery to clathrin-mediated endocytosis. A LIR motif was also detected in the clathrin heavy chain itself (6). However, it remains unclear what functional consequences this has beyond the biochemical interaction. Finally, as a third component of clathrin-mediated endocytosis that might depend on the macroautophagy machinery for its efficient recruitment to cell membrane receptors, the adaptor-associated kinase 1 (AAK1) was recently identified as an Atg8/LC3 interactor and contains predicted LIR motifs (46). AAK1 phosphorylates the μ subunit of the AP2 complex for more efficient clathrin-dependent internalization but might also facilitate clathrin-independent endocytosis (47, 48). In Atg5- or Atg7-deficient mouse DCs, MHC class I surface levels were increased, while B and T cells in the same mice showed no differences in MHC class I surface levels *in vivo* (46). This increased surface expression resulted from diminished internalization, and AAK1 was not efficiently recruited to MHC class I molecules in Atg5- or Atg7-deficient DCs. This resulted in increased CD8^+^ T cell stimulation *in vitro* and elevated CD8^+^ T cell responses to influenza A virus (IAV) and lymphocytic choriomeningitis virus infection *in vivo*, as well as improved immune control of IAV. However, not only classical MHC class I molecules are affected by diminished clathrin-dependent receptor internalization in the absence of Atg8/LC3 lipidation but also the non-classical MHC class I molecule CD1d gets stabilized on the cell surface of Atg5-deficient DCs (49). These non-classical MHC class I molecules present glycolipids to NKT cells (50). The increased CD1d surface stabilization in the absence of Atg-dependent internalization led to increased NKT cell stimulation *in vitro* and *in vivo* (49). Furthermore, the NKT cell-dependent pathogen *Sphingomonas paucimobilis* was more efficiently restricted in mice with Atg5 deficiency in their DCs. These studies suggest that Atg/LC3 lipidation assists clathrin-mediated phagocytosis by recruiting different components of the respective endocytic pathway to the cell membrane.

## Atg Proteins in Inflammatory Mediator and Antigen Release

The above-described pathways still utilize Atg proteins for lysosomal degradation, albeit not through intracellular delivery, but degradation of endocytosed cargo and surface receptors. However, as a non-catabolic function of the macroautophagy machinery, it was noted that antigen release for efficient cross-presentation on MHC class I molecules requires Atgs in antigen donor cells (51, 52). This role during unconventional secretion was first demonstrated for IAV-infected cells or tumor cells in these two initial studies. The respective vesicles, which might be related to Atg8/LC3 containing exosomes that originate from multivesicular bodies (53), can be forced to be released in higher numbers by inhibiting lysosomal degradation and to incorporate defective ribosomal products by proteasome inhibition (54, 55). Therefore, they have been coined defective ribosomal products-containing autophagosome-rich blebs (DRibbles). Moreover, they contain some TLR and NOD2 agonists to activate antigen-presenting cells, at the same time as they transfer antigen (56). These formulations have been used to vaccinate mice against a variety of tumor challenges (57–60). Thus, autophagic cargo gets released from transformed and infected cells in vesicles that can be efficiently taken up and activate antigen-presenting cells to induce antitumor immune responses.

**Figure 2:**
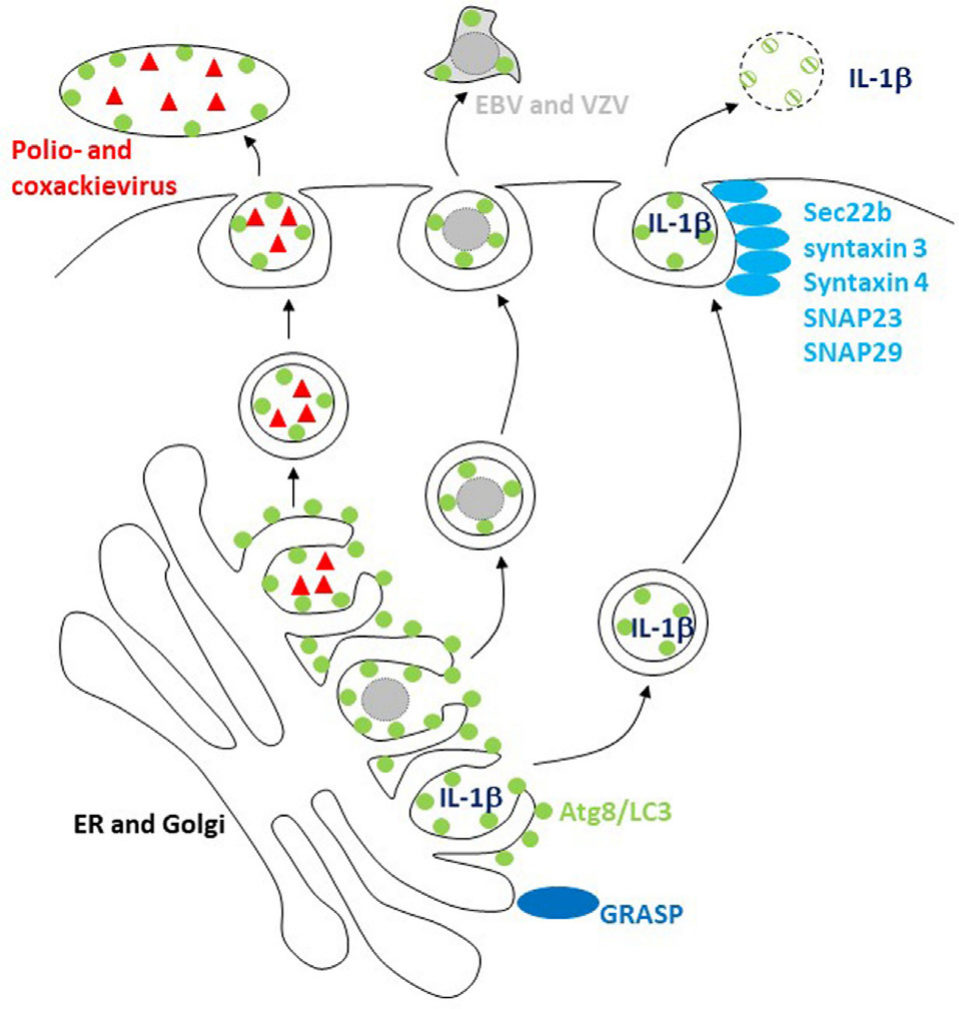
The macroautophagy machinery supports unconventional exocytosis. Atg8/LC3-conjugated membranes facilitate the release of packages of picornaviruses (poliovirus and coxsackievirus) and of herpesviruses [varicella zoster virus (VZV) and Epstein–Barr virus (EBV)]. Unconventional ER targeting signal peptide-independent secretion of caspase-processed IL-1β also required Atg8/LC3 lipidation for release. Golgi reassembly and stacking proteins (GRASPs) and the SNAREs Sec22b, syntaxin 3 and 4, and SNAPs 23 and 29 are involved in this release and fusion of Atg8/LC3-coupled membranes with the cell membrane.

These findings point toward unconventional ER targeting signal peptide-independent secretion by the autophagic machinery. Indeed, acyl coenzyme A-binding protein has been described to be secreted by yeast and ameba in an autophagy-dependent fashion (61, 62). This secretion is Golgi reassembly and stacking protein (GRASP) dependent. Similarly, the secretion of caspase-processed IL-1β is also dependent on Atgs in mammalian cells (Figure 2) (26, 27). It is worthwhile pointing out that the net outcome of Atg deficiency in myeloid cells as a major source of inflammasome-dependent IL-1β release is usually hyperinflammation (40) and that IL-1β usually leaves these cells during pyroptosis *via* gasdermin-dependent cell lysis (63, 64). However, in cells in which mature IL-1β is expressed without inflammasome activation and pyroptosis, IL-1β is released in a GRASP- and Atg-dependent fashion that involves the SNAREs Sec22b, syntaxin 3 and 4, as well as SNAPs 23 and 29 for membrane fusion (65). The cell type and physiological condition under which such Atg-dependent IL-1β secretion, however, occurs still needs to be identified. Additional cargo for this unconventional secretion pathway includes ferritin (65), HMGB1 (66), and secretory lysosomes (67). These studies suggest that Atg proteins support unconventional secretion, but how substrates are selected for this secretion versus degradation by autophagy still needs to be characterized.

## Atg Proteins in Viral Release

Viruses might be able to teach us how Atg-dependent secretion versus degradation can be regulated, because a number of them seem to harness Atg8/LC3-coupled membranes for their release (16). The first virus that was found to stabilize Atg8/LC3-associated membranes was a picornavirus, i.e., poliovirus (68). The release of poliovirus was dependent on these structures (69), and it was proposed that viral RNA replication and capsid assembly occurs in Atg8/LC3-coated double-membrane-surrounded vesicles, which then fuse with the cell membrane after acidification (70). Although poliovirus and related picornaviruses are non-enveloped, it was recently observed that they are released from cells in packages of multiple viral particles enveloped in a lipidated Atg8/LC3-positive membrane (Figure 2), topologically similar to the inner autophagosome membrane (23). Similarly, the closely related picornavirus coxsackievirus B is also released in packages that are surrounded with LC3-II-containing membranes (71). These packages might explain why coxsackievirus B spreads efficiently through cultures of cells with an intact macroautophagy machinery (72). This benefit for viral dissemination could result from protection by the surrounding Atg8/LC3-coupled membrane and its phosphatidylserine (PS) content in the outer membrane leaflet, which allows for efficient uptake by phagocytes *via* scavenger receptors that usually clear apoptotic cells (23). Indeed, ER and Golgi membranes seem to have substantial amounts of PS in both inner and outer leaflet, and sampling from this source of autophagic membranes might endow viruses with an envelope lipid composition that is beneficial for infection *via* clearance pathways for apoptotic cells (73).

Herpesviruses might also use this pathway for envelope acquisition. They acquire their second and final envelope from ER and Golgi membranes in the cytosol (74). Indeed, a γ-herpesvirus, i.e., Epstein–Barr virus (EBV), was found to stabilize Atg8/LC3-coupled membranes during lytic replication (22, 75). Loss of Atg proteins inhibited the release of infectious EBV particles (22, 75), and viral DNA was trapped in the cytosolic fraction (22). Similar to poliovirus packages, lipidated Atg8/LC3 enriched with EBV particle purification from the supernatant of virus replicating (Figure 2), but not latently EBV genome carrying cells (22). Furthermore, Atg8/LC3 could be observed in purified virus particles by immunoelectron microscopy (22). However, EBV is not the only herpesvirus that seems to use autophagic membranes. The α-herpesvirus varicella zoster virus (VZV) also exits cells with Atg8/LC3-coated membranes, and its replication is inhibited by Atg silencing (76, 77). It is worthwhile noting that apart from EBV and VZV, α- and γ-herpesviruses contain also members, namely HSV and Kaposi sarcoma-associated herpesvirus (KSHV), which instead of utilizing Atg-dependent membranes inhibit their generation (78–80). Even though these viruses are closely related, their differences on cellular tropism might dictate why HSV and KSHV rather inhibit, while VZV and EBV utilize autophagic membranes during replication.

As a last example for redirecting autophagic membranes to the cell surface, influenza A virus (IAV) infection will be discussed. IAV infection also accumulated Atg8/LC3-conjugated membranes upon infection (81–83). Autophagosomes did not fuse with lysosomes upon IAV infection, but instead accumulated around the nucleus, and Atg8/LC3-positive membranes were rerouted to the cell membrane (81, 82). Both accumulation and rerouting are caused by matrix protein 2 (MP2) of IAV. MP2 contains a LIR motif that is required for Atg8/LC3-positive membrane accumulation on the cell surface (82), and MP2 proton channel activity contributes to the block in lysosome fusion and perinuclear accumulation of autophagosomes (83). This autophagic membrane rerouting provides sufficient membranes for filamentous budding of IAV, which increases the stability of the resulting IAV particles, possibly *via* changing the membrane composition of the IAV envelope (82). However, Atg8/LC3 itself is not incorporated into infectious IAV particles. Nevertheless, Atg proteins seem to be used by viruses to select membranes for their envelopes to improve viral transmission.

## Conclusion and Outlook

Macroautophagy uses a membrane remodeling machinery of Atg proteins to form autophagosomes around cargo that is destined for lysosomal degradation. This machinery acts in a modular format for activation of PI3K activity by phosphorylation *via* ULK1, for PI3P deposition on membranes, which is then used for the recruitment of the Atg8/LC3 lipidation machinery. These modules of autophagosome formation can be used in other membrane remodeling pathways, in which substrates need to be recruited to lipid bilayers *via* PI3P or Atg8/LC3. For example, LAP uses only the PI3K and Atg8/LC3 lipidation modules. Future research will need to unravel how the Atg modules are distributed to the different tasks and how the resulting membrane marks result in different cell biological outcomes. A detailed understanding might allow us to harness Atg proteins for therapeutic approaches against infectious diseases, cancer, neurodegeneration, hyperinflammatory diseases, and aging.

## Author Contributions

The author confirms being the sole contributor of this work and approved it for publication.

## Conflict of Interest Statement

The author declares that the research was conducted in the absence of any commercial or financial relationships that could be construed as a potential conflict of interest.
